# Evidence of SARS-CoV-2 Antibodies and RNA on Autopsy Cases in the Pre-Pandemic Period in Milan (Italy)

**DOI:** 10.3389/fmicb.2022.886317

**Published:** 2022-06-15

**Authors:** Alessia Lai, Stefano Tambuzzi, Annalisa Bergna, Alessio Battistini, Carla Della Ventura, Massimo Galli, Riccardo Zoja, Gianguglielmo Zehender, Cristina Cattaneo

**Affiliations:** ^1^Department of Biomedical and Clinical Sciences, University of Milan, Milan, Italy; ^2^Department of Biomedical Sciences for Health, Institute of Forensic Medicine, University of Milan, Milan, Italy

**Keywords:** COVID-19, coronavirus disease, SARS-CoV-2, Milan, Italy, autopsy, cadaveric blood

## Abstract

In this study, we analyzed blood samples obtained from 169 cadavers subjected to an autopsy from 1 October 2019 to 27 March 2020. The presence of anti-severe acute respiratory syndrome coronavirus 2 (anti-SARS-CoV-2) antibodies was searched by lateral flow immunochromatographic assay (LFIA) and ELISA tests and the SARS-CoV-2 RNA was tested in blood and available lung tissues by real-time PCR (RT-PCR) and droplet digital PCR (ddPCR). Five cases resulted in positives at the serological screening for anti-SARS-CoV-2. Three results were weakly positive for IgM while only one showed strong reactivity for IgG antibodies. The fifth subject (who died in December 2019) resulted positive for the ELISA test. The detection of SARS-CoV-2 RNA resulted in positive only in the blood and lung tissues of such cases. These data suggest that cadaveric blood may be a suitable substrate for the assessment of SARS-CoV-2 infection; moreover, they extend the observations of sporadic cases of SARS-CoV-2 infection in North Italy prior to the first confirmed cases.

## Introduction

At the end of December 2019, the novel severe acute respiratory syndrome coronavirus 2 (SARS-CoV-2) causing serious pneumonia was identified in Wuhan, Hubei Province, China (Jin et al., 2020). The coronavirus disease 2019 (COVID-19) rapidly spread worldwide, and the World Health Organization (WHO) declared pandemic status in March 2020. The first laboratory-confirmed Italian COVID-19 case was identified in Lombardy on 21 February 2020, in a 38-year-old man who had no history of possible contact with positive cases in Italy or abroad. Within a few days, additional cases of COVID-19 and critically ill patients were recorded in the surrounding area. Soon, several cases were identified in other Italian regions, mostly in the northern area. Based on early phylogenetic studies, it was hypothesized that the virus had been circulating in Italy since late January 2020 (Zehender et al., 2020; La Rosa et al., 2021; Micheli et al., 2021). However, the rapid spread, the large number of patients requiring hospital admission and treatment in intensive care units already at the beginning, as well as the duration of the pandemic, suggested that the arrival of the virus and its circulation in Italy in a less symptomatic form could be anticipated. Some recent studies have investigated this hypothesis using serological or molecular methods. A seroepidemiological study of patients with lung cancer found anti-SARS-CoV-2 antibodies in the plasma samples of 12% of patients coming from different Italian regions beginning in September 2019 (Apolone et al., 2021). Other authors have analyzed oropharyngeal swabs performed on a pediatric population through PCR technique, starting in September 2019, identifying a positive case for SARS-CoV-2 RNA in a swab collected on 5 December 2019 from a child with rhinitis and measles-like skin rash (Amendola et al., 2021). However, other authors reported the absence of the circulation of SARS-CoV-2 in Rome during the pre-pandemic period (Capalbo et al., 2020). Environmental assessments were also performed to search for SARS-CoV-2 by real-time PCR (RT-PCR) technique by analyzing wastewater samples collected in October 2019 from five treatment plants in three cities and regions of northern Italy (Milan/Lombardy, Turin/Piedmont, and Bologna/Emilia Romagna). Some samples tested positive and, in detail, the first dating back to 18 December 2019 in Milan and Turin and to 29 January 2020 in Bologna (La Rosa et al., 2021).

To date, no studies aimed at searching for evidence of the circulation of SARS-CoV-2 in the pre-pandemic period have been conducted on autopsy cases. We wanted, therefore, to address this specific topic by analyzing blood samples collected from cadavers subjected to autopsy at the Institute of Forensic Medicine in Milan. Being such a city, the regional capital epicenter of the first-ever outbreak of COVID-19 in Italy (Calati et al., 2021), the ultimate purpose was to investigate the existence of further evidence of the human circulation of SARS-CoV-2 before the start of the first officially reported case.

## Materials and Methods

### Setting

The city of Milan and its hinterland have only one Institute of Forensic Medicine. All deaths for which it is deemed necessary to perform a pathological or a forensic autopsy, therefore, come to our attention. Only deaths occurring in hospitals may be an exception since in such cases the pathological autopsy can be performed directly on site. For these reasons, and in consideration of the populousness of Milan, the Institute of Legal Medicine has a vast people influx, almost representative of the entire population.

### Procedure

At the Institute of Forensic Medicine in Milan, all the samples taken over the past 5 years from bodies subjected to an autopsy are regularly stored. Among these, there are also cardiac or femoral blood samples that have been collected for carrying out any future toxicological analysis. We, therefore, selected all the autopsy cases from 1 October 2019 to 27 March 2020 for which blood samples had been collected. Upon the autopsy, cardiac blood is collected directly from the heart chambers and femoral blood from the femoral artery; blood samples are introduced into 10 ml plastic tubes and stored in the refrigerator at an average constant temperature of 3–4°C. An aliquot of 3 ml was taken from each tube and stored at −20°C for virological investigations.

Upon autopsy, visceral samples are also collected for any future histological analysis. In particular, lung fragments are routinely sampled.

To evaluate the performance of the protocol used to detect antibodies and/or viral RNA in cadaveric blood, we also included four samples from subjects who died from COVID-19, between March and December 2020. Cadaveric blood was obtained as previously reported.

### Serological and Molecular Analyses

Microbiological analyses were performed at the Laboratory of Infectious Diseases of the University of Milan at “Luigi Sacco” Hospital, Milan.

Three investigations were carried out on each sample and positive controls: (i) rapid serological test, (ii) ELISA test, and (iii) RT-PCR test.

All the blood samples obtained from study subjects and four controls were subjected to a screening for anti-SARS-CoV-2 antibodies.

Two serological tests were adopted to overcome the possible poor sensitivity of these methods to cadaveric blood. Additionally, the search for the RNA of the virus using RT-PCR was carried out on all samples. Given the low sensitivity of the RNA detection in blood and the lack of stored swabs, in subjects with the availability of the autoptic lung tissues, we searched SARS-CoV-2 RNA in parallel in blood and lung tissue.

The presence of IgM/IgG against SARS-CoV-2 was qualitatively determined on blood samples (20 μl) by a lateral flow immunochromatographic assay (LFIA) against the Nucleocapsid protein (COVID-19 IgG/IgM Rapid Test, Prima Lab, Balerna, Switzerland) and using a SARS-CoV-2 Total antibodies detection ELISA test (Beijing Wantai Biological Pharmacy Enterprise, Beijing, China), in accordance with the manufacturer's protocols. The declared performance of the lateral flow immunoassay for IgG was: specificity 98.0%, sensitivity 100%, and accuracy 98.6%; for IgM: specificity 96.0%, sensitivity 85.0%, and accuracy 92.9%. The ELISA test, based on the principle of a double-antigen sandwich detecting plasma/serum total antibodies against SARS-CoV-2 spike protein (the receptor-binding domain), had a sensitivity and specificity between 93 and 98% and 99 and 100%, respectively.

Blood samples were previously diluted 1:2 with phosphate-buffered saline (PBS) and the mixture was continuously rotated overnight at 37°C before being centrifuged at 14,000 rpm for 15 min.

Droplet digital PCR (ddPCR) was performed in subjects who tested positive or with an uncertain result at RT-PCR.

RNA was manually extracted from blood samples with the QIAamp Viral RNA kit (QIAGEN, Venlo, Netherlands) according to the manufacturer's instructions. RNA was eluted in 50 μl of water and used as the template for RT-PCR performed using qPCR Luna^®^ Universal One-Step RT-qPCR (New England BioLabs, USA) and SARS-CoV-2 (2019-nCoV) CDC qPCR Probe Assay (Integrated DNA Technologies Inc., USA) kits were used. The assay targets regions N1 and N2 of the SARS-CoV-2 nucleocapsid gene as well as the housekeeping gene Human RNase P (RP).

Once results on all 169 samples were obtained, for all the individuals whose results were positive either for antibodies or viral RNA, lung specimens were also tested when available. They were each cut with a thickness of up to 20 μm and were processed using the RNeasyDSP FFPE kit (QIAGEN, Venlo, Netherlands) for sample homogenization and RNA extraction according to the manufacturer's instructions.

Droplet digital PCR was performed using a SARS-CoV-2 ddPCR kit (Bio-Rad, Pleasanton, CA, USA) for the detection of SARS-CoV-2 RNA in the lung tissues and the blood. The assay targets regions N1 and N2 of the SARS-CoV-2 nucleocapsid gene as well as the housekeeping gene Human RNase P (RP). Negative and positive droplets were analyzed using the QX200 Droplet Reader and the QuantaSoft software (Bio-Rad v. 1.7.4, Pleasanton, CA, USA). Quantification was finally expressed in copies/reaction (20 μl).

All samples were tested using two replicates.

### Ethical Approval

The samples taken from the subjects enrolled in this study and the subsequent laboratory analyses were all performed by Italian law.

## Results

### Subject Characteristics

A total of 169 cases were enrolled by selecting all autopsy cases from 1 October 2019 to 27 March 2020 for which at least one blood sample had been collected and stored. They were all in a good state of preservation, except for 12 cases (7%), which were characterized by active decay or an advanced state of decomposition. The temporal distribution pattern, month by month, of all the enrolled deceased was as follows: 33 cases in October 2019, 34 cases in November 2019, 38 cases in December 2019, 30 cases in January 2020, 24 cases in February 2020, and, finally, 10 cases in March 2020. Overall, from an epidemiological point of view, the male gender was more prevalent with 136 cases (80.5%), compared with the female one with 33 cases (19.5%). The youngest victim was a 12-year-old girl, while the oldest one was a 99-year-old. The mean age of the victims was 53 years. By dividing all cases into decades, we observed that there were 3 victims between 10 and 19 years (m = 2 and f = 1), 16 victims between 20 and 29 years (m = 13, f = 3), 15 victims between 30 and 39 years (m = 10, f = 5), 36 victims between 40 and 49 years (m = 28, f = 8), 40 victims between 50 and 59 years (m = 36, f = 4), 30 victims between 60 and 69 years (m = 27, f = 3), 13 victims between 70 and 79 years (m = 10, f = 3), 13 victims between 80 and 89 years (m = 9, f = 4), and finally 3 victims between 90 and 99 years (m = 1, f = 2). Italian nationality was by far the most prevalent (142 cases), followed by other smaller ethnic groups, both European and non-European. Out of all the 169 cases enrolled, in 132 cases the biological substrate available was found to be cardiac blood, and in the remaining 37, femoral blood. The four controls were patients who died of COVID-19.

### Serological and Molecular Investigations

Moreover, five out of the total 169 blood samples resulted in positive either for anti-SARS-CoV-2 antibodies or SARS-CoV-2 RNA as shown in the Table 1, in which the main epidemiological and forensic data of the corresponding victims are also reported. The rapid serological test revealed four positive blood samples, three of which were weakly positive for anti-SARS-CoV-2 IgM but negative for IgG (#1, #2, and #4) and 1 case (#5) showed strong reactivity for IgG antibodies and was negative for IgM. At the ELISA test, a single positivity was observed in a subject (#3) that was negative for the rapid test (Table 1). Out of all these five subjects, lung specimens were available in only two cases (#3 and #5, which are highlighted in gray color in the table).

**Table 1:** Epidemiological and forensic data of the 5 samples resulted positive to at least one virological analysis.

**Case N**.	**Sex**	**Age**	**Death date**	**Autopsy date**	**Cause of death**	**Manner of death**	**Biological material**	LFIA*	**ELISA**	**SARS-CoV-2 RNA Blood**	**SARS-CoV-2 RNA Lung**
1	M	36	19.10.19	24.10.19	Hanging	Suicide	Cardiac blood	IgM	Neg	Neg	
2	M	57	24.10.19	28.10.19	Hanging	Suicide	Cardiac blood	IgM+	Neg	Neg	
3	M	58	09.12.19	16.12.19	ACI^†^	Natural	Femoral blood	Neg	Pos	Pos	Pos
4	F	50	09.12.19	17.12.19	ACI	Natural	Cardiac blood	IgM+	Neg	Neg	
5	M	54	14.01.20	16.01.20	ACI	Natural	Cardiac blood	IgG++	Neg	Neg	Neg

*LFIA, lateral flow immunochromatographic assay.

*^†^ ACI, Acute Circulatory Insufficiency*.

All four COVID-19 positive controls tested positive for serological tests (both the rapid test and the ELISA); in particular, three samples resulted positive for both IgG and IgM antibodies and one only for IgG antibodies.

The search of SARS-CoV-2 RNA by RT-PCR resulted in negative in all seropositive subjects but one (#3) who was simultaneously positive in blood and lung, showing at the ddPCR 38.3 and 282.9 RNA copies/reaction, respectively. The subject positive for IgG antibodies (#5) showed negative RT-PCR both in the blood and lung. None of the four COVID-19 positive controls showed viral RNA in the blood by RT-PCR.

By integrating such findings with the epidemiological-forensic data, it was observed that out of the three IgM weakly positive cases, two of them (#1 and #2) referred to subjects who died in the second half of October 2019, and the third one (#4) to a subject who died in December 2019. Case #3, which was positive for SARS-CoV-2 antibodies and RNA, referred to a subject who died in the first half of December 2019 (9 December 2019). Finally, the last case (#5), which tested IgG positive referred to a subject who died in mid-January 2020. In addition, we point out that all five of these subjects had been seen alive the day before the discovery and that, therefore, at the time of the autopsy (and sampling), they were in a good condition of preservation. Finally, in two cases, the cause of death was identified as hanging, and in the remaining three, it was a generic acute circulatory insufficiency. We use such terminology when, at the end of the autopsy, no pathological elements that can be peremptorily identified as a cause of death have been detected.

## Discussion

Severe acute respiratory syndrome coronavirus 2 (SARS-CoV-2) was discovered in December 2019 in China and has since spread widely in many countries. The first SARS-CoV-2 cases reported in Italy were two Chinese tourists who fell ill in January after flying in from Wuhan, while the first autochthonous COVID-19 cases in Italy were reported in the town of Codogno in the Lombardy region on 21 February 2020 and in the Veneto region on the same day.

The timing of the first introduction of the virus is of epidemiological relevance, for the tracking and mapping of COVID-19 spread. It has been hypothesized, based on some evidence, that a silent circulation of the virus may have preceded the onset of the epidemic in European countries for a more or less long time. In France, SARS-CoV-2 was found in a stored respiratory sample from a patient hospitalized in December 2019, suggesting the earlier presence of the virus than previously thought (Deslandes et al., 2020). More recently, a serosurvey study of a general population-based cohort suggested an occurrence of the infection in the country as early as October 2019 (Carrat et al., 2021). Phylogenetic studies on samples collected in Italy suggested a possible cryptic circulation of SARS-CoV-2 in the country sometime before the epidemic became evident (Lai et al., 2020; Zehender et al., 2020). Moreover, the presence of SARS-CoV-2 RNA was reported in untreated wastewater samples collected in the Milan area as early as mid-December 2019 (Micheli et al., 2021).

Our study was aimed to investigate the presence of SARS-CoV-2 in the Milan area in an unselected series of autopsy cases that occurred from October 2019, using for the first time stored blood samples, obtained post mortem, for serological studies.

The SARS-CoV-2 antibody seroprevalence was tested using a lateral flow immunochromatographic assay for serum IgG or IgM directed against the nucleocapsid protein and an ELISA test for detecting antibodies against the receptor binding domain of SARS-CoV-2 spike protein. Both assays showed high sensitivity and specificity as reported in previously published studies (Milazzo et al., 2021; Valenti et al., 2021). The performance in detecting anti-SARS-CoV-2 antibodies of the LFIA and ELISA tests employed in this study was previously estimated by us, obtaining a specificity of 0.98 and 0.99, and a sensitivity of 0.94 and 0.97, respectively (Zehender et al. personal data unpublished).

In this study, LFIA detected only a weak positivity for IgM in three subjects and a single strong positivity for IgG antibodies in another one, while another case tested positive for ELISA, but negative for LFIA. On the contrary, the four samples obtained by subjects who died from COVID-19 in the early times of the Italian epidemic, which were included in the study as control specimens, tested positive with both methods.

Previous studies on the detection, in post mortem, collected blood, of antibodies against other viruses, such as HBV, HCV, and HIV, showed a different frequency of false positive or negative results at the Elisa test, suggesting that blood samples taken from non-heart-beating donors perform worse than samples from living subjects (Padley et al., 2005). To our knowledge, there is no other experience with the use of serological testing for SARS-CoV-2 on a series of unselected post-mortem blood samples. The performance of these tests on this kind of sample must, however, be considered with caution.

Real-time PCR testing resulted in negatives in all samples but one, the ELISA seropositive subject #3, who showed a small amount of SARS-CoV-2 RNA in the blood and lung tissues. SARS-CoV-2 has been detected in specimens from multiple sites (Peng et al., 2020; Wang et al., 2020; Zheng et al., 2020), including blood and plasma. In blood, its frequency ranges from 1 to 73% (Chen et al., 2020; Hogan et al., 2021) in patients with COVID-19, and it seems to be significantly associated with adverse clinical outcomes (Hagman et al., 2021). However, the viremia is transient and infrequent, especially in paucisymptomatic or asymptomatic cases of COVID-19 (Andersson et al., 2020; Owusu et al., 2021). On the contrary, a recent work demonstrated that SARS-CoV-2 RNA could persist for longer periods in the lung (Caniego-Casas et al., 2022).

Although in most cases we analyzed fresh bodies with no relevant post-mortem interval (PMI), we cannot exclude that the small number of SARS-CoV-2 positive samples could have been underestimated due to post-fatal phenomena and the long storage period of samples, thus explaining the apparent absence of reactivity to SARS-CoV-2 antibody and RNA in samples obtained between February and March (a total of 24 in February and 10 in March 2020). On the other hand, the seroprevalence of SARS-CoV-2 antibodies in healthy adults in Milan at the beginning of the epidemic (between the end of February and March) was <10%, reaching values between 2.4 and 9.0% only at the end of March (Valenti et al., 2021)**.**

Comparative data indicated that ddPCR represents an accurate method for SARS-CoV-2 detection in low viral copies with higher sensitivity than the classical RT-PCR (Park et al., 2021). In line with this observation, in our case file, ddPCR was able to detect the presence of SARS-CoV-2 RNA at a small copy number in the lung of the subject #3 (positive also at the RT-PCR). This finding also confirmed the persistence of SARS-CoV-2 viral RNA in the lung after a short PMI (Musso et al., 2021). Comparing the results obtained from different samples of cases #3 and #5, a recent infection—interesting also the lungs—could be hypothesized for case #3, considering that LFIA frequently tests negative before 10 days from the onset of symptoms, while ELISA often tests positive earlier.

On the contrary, a long time passed from infection for subject #5 given the strong positive result for IgG antibodies and the SARS-CoV-2 RNA negativity in the blood and the lung.

Notably, our work demonstrates the presence of COVID-19 infection at the time of death, despite the reported cause of death not being associated with the virus infection.

To the best of our knowledge, this is the first study that shows that cadaveric blood may be a suitable substrate for the assessment of SARS-CoV-2 infection, even if further studies are needed. Moreover, it demonstrates in a large autopsy case series the presence of the virus in Italy before February 2020. In line with other reports (Amendola et al., 2021), our data indicated that the first SARS-CoV-2 positive case dated December 2019, while the detection of positivity only to IgM test by rapid LFIA in October 2019, being within the lower limit of the specificity rate of the test, possibly represents nonspecific signal or cross-reaction with antibodies against other coronaviruses. Recent phylogenetic studies have suggested that the introduction of SARS-CoV-2 in Europe dated to January and early February 2020 (Lai et al., 2020; Alteri et al., 2021; Nadeau et al., 2021). Nevertheless, this date corresponds to the origin of the SARS-CoV-2 clade 20A, characterized by the amino acid substitutions D614G in S protein and P314L in ORF1b, and corresponding to an early Italian cluster observed also in other world countries (Lai et al., 2020; Zehender et al., 2020). This strain formed the earliest large cluster of infections in northern Italy, and then spread rapidly throughout Europe and worldwide, but other introductions of different strains have been documented (Lai et al., 2021). Therefore, we cannot exclude, in earlier times, before clade 20A evolution, the sporadic introduction of different viral strains, endowed with lower transmissibility and spreading potential, not giving rise to a sustained epidemic.

The COVID-19 pandemic also raised another very important issue, which is the need for infection control strategies for the safe management of clinical and forensic autopsies with suspected or confirmed COVID-19. Both the WHO (https://www.who.int/publications/i/item/infection-prevention-and-control-for-the-safe-management-of-a-dead-body-in-the-context-of-covid-19-interim-guidance) and the Centers for Disease Control and Prevention (CDC) (https://www.cdc.gov/coronavirus/2019-ncov/hcp/guidance-postmortem-specimens.html) published post-mortem guidance frequently reviewed during the pandemic. Autopsies have to be conducted in Airborne Infection Isolation Rooms (AIIRs) with a negative pressure of 9.7 air changes per hour (ACH). Doors have to be kept closed during the procedure, and the AIIR room air has to be exhausted directly outdoors, away from windows, areas of human traffic, or gathering spaces (Fineschi et al., 2020). Moreover, personal protective equipment (PPE), such as disposable gloves, FFP3 respiratory filters, goggles or protective visor, disposable long-sleeved gown or waterproof suit, and disposable overshoes, are required when performing an autopsy on suspected COVID-19 subjects (Padley et al., 2005; Hanley et al., 2020; Pomara et al., 2021). Cut-resistant gloves should also be used because of the risk of puncture. Used PPE should be appropriately disposed of in suitable containers. It is necessary to avoid contact with the face and mouth and to wash hands after the autopsy (Finegan et al., 2020). Recently, the analysis of environmental swabs for SARS-CoV-2 in an autopsy room has demonstrated that autopsy is a safe procedure with a minimal infection risk for all subjects involved when appropriate strategies are adopted (Pomara et al., 2021). Therefore, the autopsy of bodies with confirmed COVID-19 should be considered a safe procedure, and as such, its practice should be encouraged.

This study has some limitations. First, the design was an autopsy study with limited clinical information regarding the status of subjects before death. Second, no nasopharyngeal swab samples were available and for this reason, we were able to analyze only blood samples for all cadavers, and for SARS-CoV-2 positive samples, only lung tissue was considered, excluding other anatomical sites. Despite the high reliability of the molecular methods for the detection of the virus, we cannot exclude the limited sensitivity and specificity of the serological tests performed on cadaveric blood.

In particular, although the positivity found in LFIA in four cases is considered probably nonspecific, the prevalence of false positivity in cadaveric blood does not differ from that observed in blood samples obtained from living donors (Valenti et al., 2021) and does not exclude their use in pre-screening in future studies on series of samples obtained post mortem. In conclusion, our data suggest that retrospective analysis of specimens collected from autopsy series can offer valuable data for the temporal reconstruction of the early stages of the COVID-19 pandemic.

## Data Availability Statement

The original contributions presented in the study are included in the article, further inquiries can be directed to the corresponding author.

## Author Contributions

AL, ST, GZ, and CC conceived and designed the study. ST, ABa, RZ, and CC collected samples from cadavers. AL, ABe, and CD performed microbiological analyses. AL, ST, GZ, and CC wrote the first draft of the manuscript. All authors contributed to manuscript revision, read, and approved the submitted version.

## Funding

This research was supported in part by Ministero dell'Università e della Ricerca PRIN202022GZEHE_01. The funding sources had no role in the study design, data collection analyses, interpretation or writing of the report.

## Conflict of Interest

The authors declare that the research was conducted in the absence of any commercial or financial relationships that could be construed as a potential conflict of interest.

## Publisher's Note

All claims expressed in this article are solely those of the authors and do not necessarily represent those of their affiliated organizations, or those of the publisher, the editors and the reviewers. Any product that may be evaluated in this article, or claim that may be made by its manufacturer, is not guaranteed or endorsed by the publisher.
